# Effects of Immediate or Delayed Estradiol on Behavior in Old Menopausal Macaques on Obesogenic Diet

**DOI:** 10.1155/2018/1810275

**Published:** 2018-09-27

**Authors:** Kristine Coleman, Nicola D. Robertson, Adriane Maier, Cynthia L. Bethea

**Affiliations:** ^1^Division of Neuroscience, Oregon National Primate Research Center, Beaverton, OR 97006, USA; ^2^Division of Comparative Medicine, Behavioral Sciences Unit, Oregon National Primate Research Center, Beaverton, OR 97006, USA; ^3^Division of Reproductive Sciences, Oregon National Primate Research Center, Beaverton, OR 97006, USA; ^4^Department of Obstetrics and Gynecology, Oregon Health and Science University, Portland, OR 97201, USA

## Abstract

Macaques have served as effective models of human disease, including pathological processes associated with obesity and the metabolic syndrome. This study approached several questions: (1) does a western-style diet (WSD) contribute to sedentary behavior or is sedentary behavior a consequence of obesity and (2) does estradiol (E) hormone therapy offset WSD or ameliorate sedentary behavior? We further questioned whether the timing of E administration (immediately following hysterectomy, ImE; or after a 2-year delay, DE) would impact behavior. Focal observations were taken on the animals in social housing over a period of 2.5 years before and after initiation of the WSD and hysterectomy. In addition, anxiety was assessed through the Human Intruder and Novel Object Tests. All animals gained weight, but ImE delayed the time to maximum weight achieved at 18 months. Over the course of the study, ImE-treated monkeys spent more time “alone” and less time in “close social” contact than placebo-controls. The DE-treated monkeys were not different from placebo-controls in these 2 outcomes. The placebo-control group exhibited more “self-groom” behavior, an indicator of anxiety, than did the ImE-treated group, and DE-treated animals approached levels observed in the ImE-treated animals. All animals exhibited an increase in “consume” behavior over time with no statistical difference between the groups. By the end of the protocol, the placebo-control group exhibited less activity compared to ImE + DE-treated animals combined. Animals also showed increased anxiety after starting on the WSD in the Human Intruder Test and the Novel Object Test. In summary, the data indicated that WSD per se promoted increased consummatory behavior, sedentary behavior, and anxiety-type behaviors, whereas ImE promoted activity. Thus, WSD may precipitate the behaviors observed in humans who then become obese, sedentary, anxious, and socially isolated. ImE replacement ameliorates some of these behaviors, but not all.

## 1. Introduction

Middle-aged women today face two major health issues, obesity and menopause, both of which precipitate a number of health problems and a decline in the quality-of-life indices. The symptoms of menopause and long-term physiological deterioration may be ameliorated by proper hormone therapy. However, due to the overgeneralization of results from the Women's Health Initiative (WHI), many women and their physicians are still concerned about its use. The WHI administered conjugated equine estrogens to women who were on average 10–12 years past menopause [1, 2]. Nonetheless, the use of estradiol-17*β* (E) administration during the perimenopause period has yielded positive outcomes with respect to mood [3, 4], cognition [5], carotid intima-media thickness (CIMT; [6]), metabolism and body composition [7], lung function [8], and immune function [9] (including multiple sclerosis [10]), compared with no treatment or delayed treatment. Still, the outcomes of clinical trials with E (and E conjugates) vary from trial to trial, whereas the effects of E replacement in laboratory animal studies have been robust and repeatable. A major factor in the difference between the human and animal studies is diet. Unlike the typical American diet, laboratory animal chow is low in fat and refined sugar, but high in micronutrients.

A large portion of the US population eats a “western-style diet” (WSD) that is high in fat and refined sugar. Two-thirds of the US population is overweight or obese [11], and 114 million people exhibit symptoms of pre diabetes or frank diabetes (American Diabetes Association). Weight gain and changes in body morphology often accompany menopause [12]. Unfortunately, today many women enter menopause already obese. As obese women enter menopause, an obvious speculation is that their risk for disease could increase over the presence of either risk factor alone [13].

Obesity plays a role in metabolic disease, which exhibits a constellation of symptoms including large waist circumference or elevated waist-to-hip ratio, elevated fasting serum glucose and serum triglycerides, abnormal cholesterol, insulin resistance, hypertension, and depression. Metabolic disease is clearly linked to type 2 diabetes and cardiovascular disease. Low activity levels and a sedentary lifestyle contribute significantly to the risk of developing metabolic syndrome. Notably, menopause is one of the greatest risk factors for developing metabolic disease. E is also anorexic, and its loss at menopause promotes weight gain.

Although lifestyle modification is the preferred treatment for metabolic disease, well-established coping behaviors, such as overeating and a sedentary lifestyle, are extremely difficult to change in humans. Similarly, in macaques, lack of exercise and stress, such as that caused by lack of conspecific contact, can also contribute to weight gain. Moreover, age and female sex also increase weight if housing is the same [14].

Based upon the above information, we wondered whether hormone therapy would maintain its beneficial effects in women if they were eating WSD and/or metabolically compromised. Nonhuman primates have provided important information related to human reproduction and cardiovascular physiology. For example, in surgically menopausal cynomolgus macaques on a high fat-high sugar diet, E administration prevented the development of atherosclerosis observed in placebo-controls [15]. To further explore this question in multiple systems, we established a colony of old surgically menopausal (ovohysterectomized, OvH) WSD-fed rhesus macaques. In addition, we sought to model single hormone therapy administered during the perimenopause with E replacement immediately upon OvH (ImE) versus E administered long after menopause (as in the WHI) with E replacement 2 years after OvH (DE; 2 monkey years = 6–8 human years). We have reported that WSD blunted or abolished the positive effects of immediate E replacement (ImE) on gene expression in the serotonin neural system [16], in circadian activity [17], and in a large number of metabolic parameters [18–20]. All of the monkeys gained weight, although ImE delayed the increase; by the end of the study, all monkeys reached the same average higher weight. The same pattern was observed in the percent of truncal fat, and in the area under the insulin and glucose curves generated from IVGTT at 6-month intervals. Rhesus macaques appear resistant to atherosclerosis [21, 22], unless the diet contains high cholesterol and 40% of calories from peanut oil [23]. Likewise, atherosclerosis was absent in coronary arteries of our WSD-fed rhesus macaques, but preliminary data suggest that WSD caused an expansion between elastin lamellae in coronary arteries (carotid intima-media thickness, CIMT), and E administration had no effect. CIMT leads to stiffness and elevated blood pressure. Finally, the group with E administration after 2 years on WSD (DE) was similar to the placebo group in most metabolic outcomes.

To determine if old, surgically menopausal, WSD-fed rhesus macaques modeled human behavior, as well as show differences in behavior in the presence or absence of E replacement, we performed longitudinal focal observations and longitudinal provoked anxiety tests on all of the animals. We hypothesized that ImE would promote increased activity and that the social housing would enable more exercise. Herein, we report the results of the behavioral observations. In addition, we comment on the process of socially housing old female rhesus macaques that were previously housed in different conditions.

## 2. Materials and Methods

The ONPRC animal care program is compliant with the laws and regulations of the United States Animal Welfare Act and is accredited by AAALAC-International. The ONPRC Institutional Animal Care and Use Committee approved this study.

### 2.1. Subjects

Twenty-eight aged (at least 17 years of age) female rhesus macaques (*Macaca mulatta*) were potential subjects for this study. The study was intended to run for 3 years, at which time the majority of monkeys would be 20 years old. Age-related pathologies manifested, and so the study was terminated after 2.5 years. Monkeys were fed standard chow (Lab Diet, Inc., St. Louis, MO) until the start of this study. After 3 months of baseline monitoring, they were switched to a “western-style diet” (WSD) also called a “typical American diet” (TAD; Lab Diet) twice daily. Regular monkey chow provides calories with 13% fat, 69% complex carbohydrates (includes 6% sugars), and 18% protein. In contrast, WSD provided calories with 36% fat, 44% carbohydrates (includes 18.5% sugars), and 18% protein. Monkeys were given fresh produce each day, and water was provided freely through automatic lixit systems. The lights were on 12 hr per day. Subjects were given enrichment such as toys, foraging devices, radio, and television to ensure their psychological health and well-being.

The monkeys were socially housed in indoor pens (approximately 3.7 m × 2.1 m × 2.1 m) situated in rooms containing up to 32 cage-housed monkeys. Each pen was located in a different room. Our goal was to place 3-4 animals in each pen; however, social introduction attempts were not always successful, and sometimes resulted in aggression. ONPRC behavioral and husbandry staff closely monitored the pens, and animals that were overly aggressive were removed from the groups. All animals were socially housed throughout the study. As shown in Table 1, the pens finally contained 2, 3, or 4 animals (*n*=24) with ongoing attrition.

Subjects were maintained on normal monkey chow during acclimation to the groups. After acclimation, subjects were fed WSD for approximately 6 weeks and then ovohysterectomized (OvH; spayed). OvH was performed because the old female macaques were to receive E for at least 30 months (2.5 years), and endometrial hyperplasia would be expected. Since older macaques are also more susceptible to cancer, OvH was used to decrease risk during the treatment period.

All animals were weighed at baseline 0 (before start of WSD), baseline 1 (before OvH), and every 3 months during the first year and every 6 months thereafter. Weight was also obtained prior to surgical procedures.

The animals were trained to run into a tunnel of smaller cages upon a hand signal. Once in the small cages, the animals could be separated for medications, injections, venipuncture, or examination.

### 2.2. Surgery

Subjects were ovohysterectomized (OvH) via the laparotomy approach by ONPRC surgical personnel. For surgeries, each animal was removed from the group, sedated with ketamine (10 mg/kg), and transported to a surgical suite. After the animals had fully recovered, they were returned to their social group.

### 2.3. Treatments

The old WSD-fed macaques received (1) placebo for 30 months, or (2) E immediately upon hysterectomy for 30 months (ImE), or (3) placebo for 24 months and delayed E for an additional 6 months (2.0 years interval; DE for 6 months). The protocol was started with 8 animals in each treatment group and 4 extra nontreated animals. The animals were organized into mixed cohorts of 8, each of which consisted of 2-3 different pens. Each pen contained animals with different treatments and different ranks. The treatment and assessment protocols were staggered 1 month by cohort to obtain all measurements on all animals in a technically feasible manner. In the first 6 months of the study, 4 animals were deemed unsuitable. They were replaced, and protocols were further staggered for the replacements. Afterwards, attrition occurred due to age-related diseases.

Estrogen was administered via Silastic capsules implanted subcutaneously in the periscapular region. One Silastic capsule (3.5 to 4.5 cm depending on metabolism; inner diameter, 0.132 in., outer diameter, 0.183 in.; Dow Corning, Mid-land, MI) was packed with crystalline estradiol (1,3,5_10-estratrien-3,17-diol, Steraloids, Wilton, NH). The implants were intended to achieve E concentrations between 70 and 100 pg/ml in the serum. An empty Silastic capsule constituted the placebo treatment.

Monkey metabolism can be highly variable, so serum E was measured every 2 months in the old WSD-fed macaques starting shortly after hysterectomy. At any time during the duration of the protocol, if serum E concentrations were higher than 120 pg/ml in an individual measurement, the capsule was replaced with a smaller capsule, which was used for that individual henceforth. When serum E levels declined below 50 pg/ml, the implants were replaced. There is a surge of E immediately after implantation that gradually declines and stabilizes; so, monitoring every 2 months enabled us to maintain the goal on average [16].

### 2.4. Behavioral Assessments

We used two methods to assess behavior and anxiety in this study: home environment assessments and provoked response tests (Human Intruder Test (HIT) and Novel Object Test (NOT)). This combined testing paradigm provided a more comprehensive picture of the emotional states of the animals than a solitary assessment method. Home environment assessments examine response to everyday, naturalistic events such as interactions with conspecifics and care staff [24]. Provoked response tests assess unconditioned response to various threatening or potentially threatening stimuli. For all tests, the observer was blind to the experimental treatment of the animals. The Observer software (Noldus Information Technology, Wageningen, Netherlands) was used to score behavior (both live and from video). The home environment assessments were performed at 5 time points, and the provoked response tests were performed at 4 time points during the study (Table 2).

The behavioral observations were obtained as part of a larger study involving these animals ([16]).

#### 2.4.1. Home Cage Behavioral Assessments

A highly trained and experienced observer took 10-min continuous focal observations [25] on the monkeys 2-3 times/week for 2-3 weeks (for a total of 60 min of observations per individual) at each time point. The observer, with whom the monkeys were familiar, entered the room and stood next to the pen for 10 min to allow the animals to acclimate to her presence. Monkeys are used to having people in their rooms and typically ignored her after a few minutes. She then began to record the behavior of the focal individual, one at a time, directly onto a laptop computer for 10 min each. To limit time of day effects, observations were taken between 12:00–3:00 pm. The order of observations was randomized, to ensure that animals had an equal probability of being observed in relationship to daily events such as afternoon feedings.  Table 3 details the ethogram of the behaviors coded in this study. Behaviors were organized into three behavioral classes: social behavior, nonsocial behavior, and events. Behaviors within the social and nonsocial behavioral class were mutually exclusive, but could co-occur with behaviors from the other class (e.g., an individual cannot be alone and touching another animal, but could be alone and stationary). Behaviors that naturally occurred in relatively short durations, such as scratches or threats, were classified as “events.” Behavioral variables were calculated as percent of time the animal engaged in that behavior (social and nonsocial behavior) or the frequency with which the behavior occurred (events).

#### 2.4.2. Human Intruder Test (HIT)

This test was designed to measure behavioral inhibition and anxiety in rhesus monkeys [26]. It assesses the behavioral response of a monkey to three stressful conditions: being alone in an unfamiliar cage, being in the presence of a human stranger whose gaze is diverted (Profile; a nonthreatening social stimulus), and being in the presence of a human stranger making direct eye contact (Stare; a threatening social stimulus).

The monkeys were temporarily removed from their social group, one at a time, brought to a dedicated behavioral testing room, placed alone in a standard monkey cage in a novel room, and videotaped from behind a one way mirror. Every animal in the group was tested on the same day, in a randomized order. The test began with a 12-minute acclimation period (alone 1). After this time period, an unfamiliar human entered the testing room and approached to 0.3 m of the cage, taking care not to make eye contact with the monkey. The human presented her facial profile to the monkey for 2 minutes (Profile). The human then left the room, leaving the monkey alone for another 2 minutes (alone 2). The human stranger re-entered the room, approached to 0.3 m of the cage, and made continuous, direct eye contact for 2 minutes (Stare). Direct eye contact is generally considered to be a threatening facial expression for monkeys. After the human intruder left, the monkey was videotaped for another 2 min (alone 3). Behaviors that were scored during this test include vocalizations, movement, and reaction to stranger, including freezing, fearful, and threatening expressions (Table 4). Behavioral variables were calculated as percent of time the animal engaged in that behavior.

#### 2.4.3. Novel Object Test

This test was designed to test the monkey's reaction to various novel objects, including an ecologically relevant novel object with reward value (i.e., a piece of unfamiliar fruit), a brightly colored toy and a rubber snake. After the alone 3 period of the HIT, the intruder re-entered the room and put various novel objects in the cage, each for 5 min. Except for the novel food, all items were removed before new objects were introduced. The novel objects were in the following order: a novel food item such as kiwi, a brightly colored bird toy, Mr. Potato Head, and a rubber snake with a piece of apple (highly desirable item) on top. Mr. Potato Head was chosen as a potentially threatening stimulus because of the large eyes.

At the end of this test, the monkey was returned to her social group. Behaviors coded from this test included the latency to inspect (approached within 3 cm), touch (intentional contact), and manipulate each item. We calculated a “Bold Score” based on an individual's reaction to the objects [27]. The scores went from 0 (low boldness; the monkey did not inspect any object within 10 seconds) to 12 (high boldness; the monkey inspected each of the items within 10 seconds).

Monkeys received their standard morning meal approximately two hours prior to the advent of the testing, to avoid the confound of hunger during the tests. Altogether, the HIT and NO tests took approximately 45 minutes.

### 2.5. Statistical Analysis

Not every animal underwent every procedure. Nine animals became ill and had to be removed from the study before 30 months; thus, there were a total of 20 animals that went through each behavioral time point (placebo *n*=6; ImE = 7; DE = 7).

For each test (home assessments, HIT, and NOT), we compared behavior across time and between treatment groups using a 2-way ANOVA followed by Sidak's multiple comparison post hoc test. One-way ANOVA or a *t*-test was used on data at the 30-month time point. Alpha values were set at 0.05. Prism v7.0 software from GraphPad was used for all analyses. Eighteen home cage behaviors were monitored, and the number of behaviors that exhibited significant differences was relatively small. Therefore, adjustment for false discovery rate (FDR) was not necessary.

## 3. Results

Eighteen individual behaviors were scored during focal observations in the home pen environment (Table 3). Table 4 outlines the behaviors recorded in the HIT. Of these behaviors, only a few were either significantly altered or warrant mention in contrast to other behaviors.

**Figure 1:**
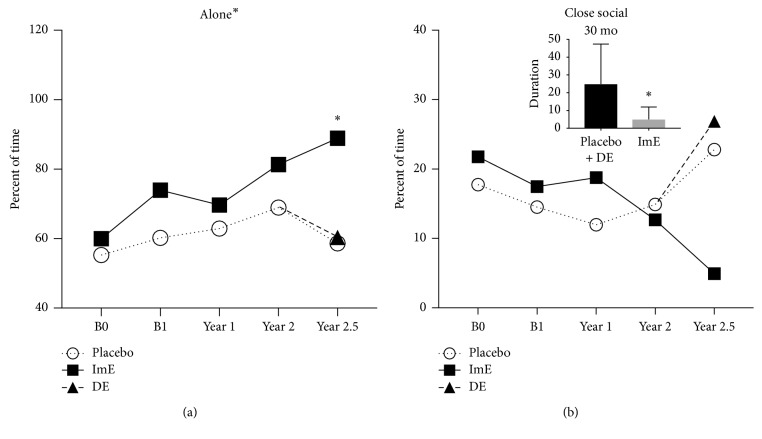
(a) The ImE-treated group exhibited higher duration of “alone” time during the protocol. In the 30-month (2.5 years) observations, there was a post hoc difference between the ImE group and the placebo or DE group (Bonferroni *p* < 0.05). (b) The ImE-treated group showed a decline in “close social” behavior over time that was statistically manifested at the 30-month (2.5 years) time point (Inset). “Close social” behavior was significantly less in the ImE group when compared with the combined placebo + DE groups (*t* test *p* < 0.017). (Title^*∗*^ different by 2-way ANOVA (*p* < 0.05); graph^*∗*^ different by post hoc test (*p* < 0.05)).

As illustrated in Figure 1, animals treated with “immediate estradiol” (ImE) at the end of the OvH surgery spent more time “alone” (not in social contact with pen mates) during focal observations as the protocol progressed (treatment, 2-way ANOVA: *F*(1, 72) = 6.622, *p*=0.012). The ImE-treated animals also showed a concomitant decrease in “close social” behavior as time “alone” increased during the study period. There was no apparent effect of DE. A combined group of placebo + DE-treated animals spent significantly more time in “close social” behavior than the ImE-treated group by the 30-month time point (*t*-test *t*(13) = 2.694; *p*=0.018).

**Figure 2:**
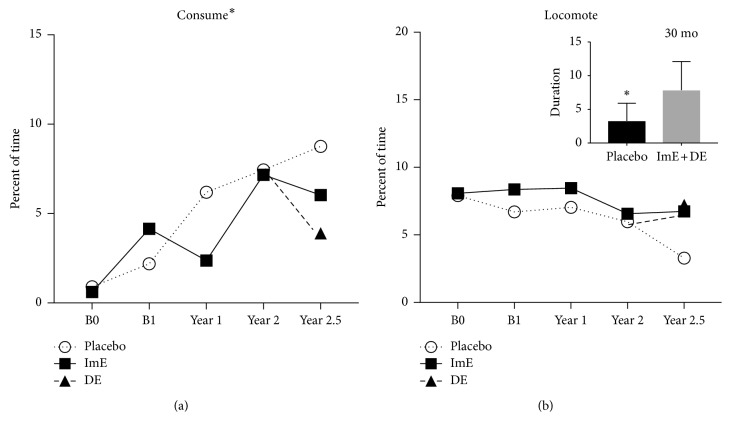
(a) There was an increase in “consume” behavior over time in all WSD-fed animals, but treatment with E had no significant effect. Comparison of the placebo group versus a combined group of ImE + DE animals was not different by *t*-test. (b) Average “locomote” behavior was significantly higher in the combined ImE + DE animals compared to the placebo-control animals at the 30-month (2.5 years) time point (*t*-test, *p* < 0.033). (Title^*∗*^ different by 2-way ANOVA (*p* < 0.05); graph^*∗*^ different by post hoc test (*p* < 0.05)).

Figure 2 shows that “consume” behavior (i.e., ingesting food and/or water) increased over time in both placebo- and ImE-treated groups (2-way ANOVA for time: *F*(1, 72) = 2.479; *p*=0.05). DE appeared to decrease “consume” behavior at 30 months, but the variance and attrition of the old animals by this time point precluded statistical significance. Concomitantly, “locomote behavior,” including large motions that would be detected by activity collars [17], exhibited a decreasing trend over time (*F*(4, 75) = 2.257; *p*=0.071). At 30 months, “locomote” in the ImE + DE combined group was higher than that in the placebo group (*t*(15) = 2.382; *p*=0.033).

**Figure 3:**
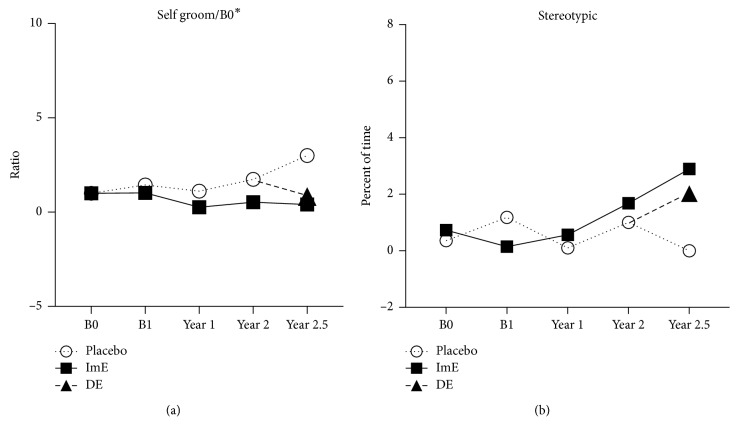
(a) Self-groom was significantly higher in the placebo animals than in the ImE treated animals (*p* < 0.045), but this was mostly due to the final time point. However, there was no difference over time. Of note, the placebo-controls showed a marked decrease in self-grooming after DE was initiated. (b) There was no difference in “Stereotypical” behavior although the DE group approached the ImE group after 6 months of treatment. (Title^*∗*^ different by 2-way ANOVA (*p* < 0.05); graph^*∗*^ different by post hoc test (*p* < 0.05)).

“Self-groom” behavior was different between placebo- and ImE-treated animals, but the groups had different baselines prior to any treatment (B0). Therefore, “self-groom” was individually normalized (divided) by B0, and the ratios were analyzed with 2-way ANOVA (Figure 3). “Self-groom” was higher in the placebo-treated animals than in the ImE-treated animals (*F*(1, 51) = 4.185; *p*=0.046). After 6 months of DE, “self-groom” behavior approached the average observed in the ImE group, suggesting that DE also decreased “self-groom” behavior.

There was no difference between the groups in stereotypical behavior (Figure 3), as determined with 2-way ANOVA (*F*(1, 78) = 1.995; *p*=0.162).

**Figure 4:**
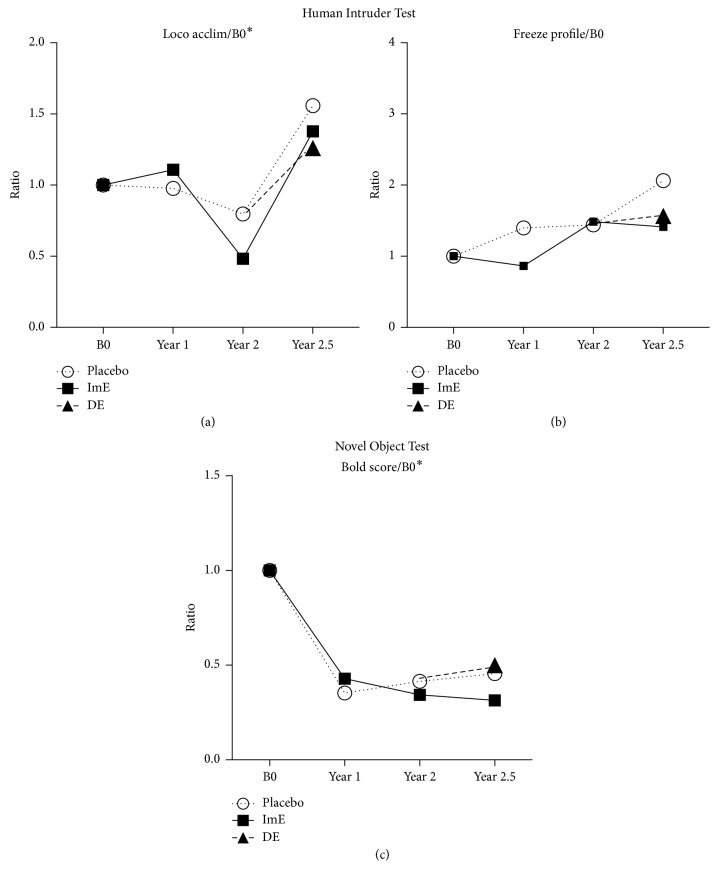
(a) Locomotion (normalized to B0) during the acclimation period was different across time (*p* < 0.041) and between treatment groups (*p* < 0.030). There was a significant difference between year 2 and year 2.5 (Tukey post hoc *p* < 0.035). (b) There was no difference between treatments or across time in freezing after normalization to B0 due to a strange human profile presented. (c)The composite “Bold Score” normalized to B0 decreased significantly in all groups by the end of year 1 (time *p* < 0.0001) and remained lower than B0 for the entire protocol. (Title^*∗*^ different by 2-way ANOVA (*p* < 0.05); graph^*∗*^ different by post hoc test (*p* < 0.05)).

Figure 4 illustrates results from the provoked anxiety tests. The animals had different personalities and thus different baseline values to start (B0), so they were individually normalized (divided) by their own B0. In the HIT, locomote-acclimate (loco acclim) in panel A reflects movement during the initial acclimation period (alone 1). “loco acclim” was not different with treatment. However, 2-way ANOVA indicated that a significant difference occurred across time (*F*(3, 62) = 4.371; *p*=0.007). The “freeze profile” in panel B indicates the length of time the animal remained completely motionless when exposed to the profile of the human intruder. There was no difference between treatment groups or across time with respect to this behavior.

At the bottom of Figure 4, panel C, the normalized “Bold Score” from the Novel Object Test is illustrated. “Bold Score” represents the individual's response to the objects. Higher scores correspond to increased propensity to inspect objects (and thus decreased anxiety toward the objects). The scores went from 0 (low boldness) to 12 (high boldness) There was a significant decrease in the normalized “Bold Score” in all animals after 1 year on WSD by 2-way ANOVA (*F*(3, 65) = 8.323; *p* < 0.0001). In other words, animals were more inhibited toward novelty after being on the WSD compared with standard chow, but there were no differences between treatment groups.

**Figure 5:**
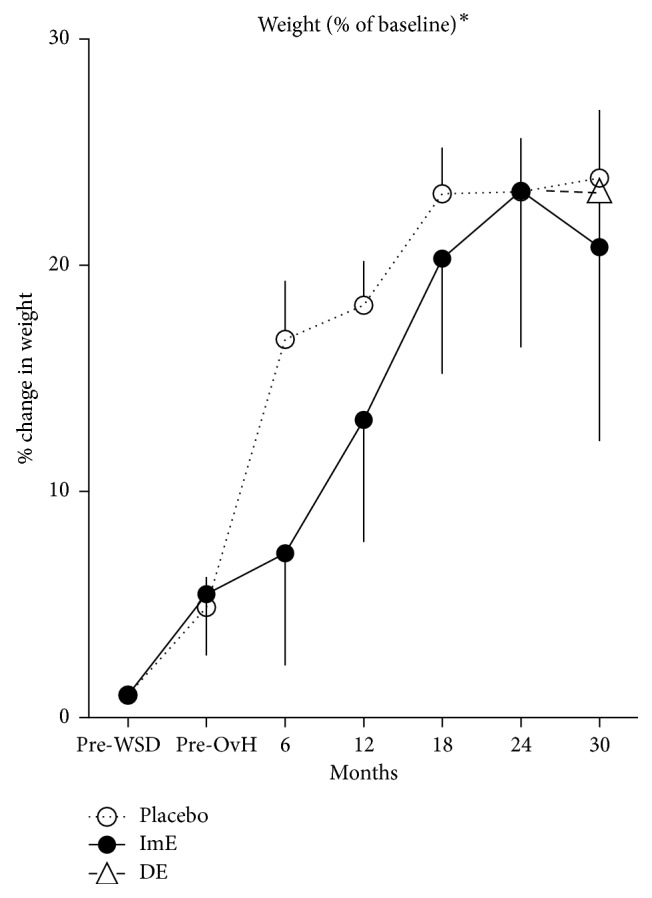
The average (±SEM) body weights as percent of baseline. Body weights increased over time in both groups and reached a similar value at 30 months. However, ImE delayed the rate of increase until 12–18 months, at which time both groups exhibited similar weights. Administration of DE had no effect on body weight. There was a significant correlation between “consume” behavior and body weight. (Title^*∗*^ different by 2-way ANOVA (*p* < 0.05).

The weights of the animals at 6-month intervals are illustrated in Figure 5, expressed as percent change from pre-WSD and analyzed with 2-way ANOVA. There was a significant effect of treatment (*p*=0.030) and time (*p* < 0.0001), but no interaction. It appeared that ImE delayed the progression to obesity, although the final weights were the same. In addition, there was a significant correlation between “consume” behavior and weight (*p*=0.003; *r*^2^ = 0.64). However, there was no correlation between rank versus “alone” (*r*^2^ = 0.1338), “close social” (*r*^2^ = 0.1693), “locomote” (*r*^2^ = 0.1543), or “consume” (*r*^2^ = 0.0339). Also, there was no effect of treatment on the final dominance rank (chi-square *p*=0.9).

## 4. Discussion

This study examined the effects of diet and E treatment on home pen behavior and on behavior during anxiety tests in rhesus macaques. To create a naturalistic environment, we sought to socially house the animals in this study. Since the goal was to model menopause, older animals (at least 17 years) were chosen for OvH. A 17-year-old female monkey is approximately equal to a 51-year-old woman (1 monkey year = 3 human years). After obtaining baseline measurements reflecting functions and behaviors when the animals were on normal monkey chow, the typical US diet, i.e. WSD, was initiated for all animals 6 weeks prior to OvH. E therapy was varied (e.g., either given immediately following OvH or after 2 years). Most of the animals were experimentally naïve and recruited from the outdoor breeding corrals. However, other animals were also used that were previously in cages, either alone or with a partner.

The greatest benefit to well-being is provided by complex social housing (groups) [28]. However, formation of the social groups was not straightforward. Several of the socialization attempts resulted in aggression, in which one or more animals had to be removed. There were factors that made these introduction attempts somewhat more challenging than most. Of the 28 animals finally assigned, 12 were used from recently reorganized outdoor breeding corrals, 12 had been in the outdoor corrals and brought inside to single housing for 1–3 months before the start of our study, and 4 had been single housed for at least 3 years. The latter 4 animals had been single-housed because they were not compatible with other female monkeys. Indeed, long periods of time housed without full contact with a conspecific can reduce future pair success in female rhesus macaques [24]. Ultimately, the 4 animals from prior single housing for 3 years were withdrawn from the study. Furthermore, all the monkeys had varying personalities, which plays a role in compatibility [29, 30]. Some of the caged monkeys were somewhat “people-oriented,” which made them ideal candidates for parts of this study (e.g., ability to work with investigators), but also made them challenging to socialize with other monkeys. While we thought that the extra space available in the pens (as opposed to cages) might help the socialization attempts, this was not true in all situations. Similar difficulties with group-housing captive rhesus macaques have been noted by several authors [29,31–33].

Another considerable factor was group stability. Group stability appeared affected by removal of an individual from the group for surgeries, which entailed recovery in a single cage for up to one week. This may confuse the remaining individuals (who could be unsure if their friend is coming back) with respect to rank. In the future, we will avoid removing only one animal at a time from the group pens for procedures that took hours or longer. Instead, all animals will be removed from the pen to single cages when one individual needs a procedure and the entire group will be returned to the pen together at the end of the day or next morning. We did not notice any stress responses from remaining animals when an individual was removed for a short period. Short protocols included temperament testing (i.e., Human Intruder and Novel Object), which is only 45 minutes in duration, venipuncture, physical exams, or getting weighed. Finally, in wild groups of rhesus macaques, males play an important part in maintaining social stability. We had no males in the group or even close to the pens to serve this function. Future studies will include an experienced, vasectomized male in each pen.

A previous study with rhesus monkeys showed important differences in weight and metabolic parameters leading to type 2 diabetes (DBT2) depending on diet, housing, age, and sex [14]. In the same study, single-cage housing was strongly linked to emerging DBT2, and the authors attributed this to exercise. Also in cynomolgus macaques, single-cage housing increased stress and decreased exercise that in turn led to obesity, metabolic abnormalities, and a host of other stereotypic and self-injurious behaviors [34, 35]. In the current study, the monkeys had room to exercise, but the WSD still led to changes in activity as described below.

With respect to surgical versus natural menopause in women, the major difference is the rate of decline of estradiol (E). With surgical menopause, the decline is abrupt, whereas with natural menopause, the decline is gradual and interspersed with random peaks of E until the ovary is completely depleted of follicles. Hormone therapy provided protection from cardiovascular events in ovary-intact perimenopausal women, but not in women who were surgically menopausal [36]. However, use of surgical menopause in this study was necessary because unlike humans, rhesus monkeys reach menopause so late in life that the risk of clinical attrition would increase greatly. This study was possible by using older monkeys and inducing menopause with surgery at the age of ∼50 human years [37].

Key aspects of this study were the longitudinal aspect of the data collected and the ability of the animals to exercise if they desired. This enabled observation of changes over time due to WSD, as well as changes due to E therapy. Also, the ability to compare outcomes before and after WSD within a reasonable time frame was a significant improvement over clinical/human studies. An important change was observed in “consume” behavior. That is, the animals spent more time eating after switching to the WSD, and neither ImE nor DE therapy altered this behavior. These observations are consistent with another study that also reported that E had no effect on feeding behavior when the monkeys were fed a highly palatable diet [38].

Concomitantly, “locomote” behavior decreased, and there was a treatment effect. By 30 months, the placebo group tended to move about their pen less than the combined ImE + DE group. Hence, WSD per se promotes increased eating and decreased activity, with a modest effect of E on locomotion. A similar pattern was detected when the movement was monitored with activity collars, but it did not reach statistical significance at the 2-year time point [17]. These data suggest that a diet high in fat and processed sugar can promote overeating and a sedentary lifestyle, which may be exacerbated after menopause. Our results are also consistent with an earlier study in which administration of a selective estrogen receptor modulator (SERM) to ovariectomized monkeys caused weight loss, reduced adiposity, suppressed food intake, and increased activity [39]. It will be very important to understand the cellular and molecular aspects of this action. For example, chronic WSD and obesity increase cytokines, and E can block the cellular actions of cytokines [40–44].

Sociality was also affected by WSD and E therapy in our study. “Time alone” was relatively stable across time in the placebo-treated animals. However, it significantly increased over time in the ImE-treated animals. Inversely, “close social” behavior decreased in the ImE-treated animals, which corroborates the increase in “time alone.” Seclusion and obesity/metabolic syndrome have been linked in a number of human studies. However, previous studies proposed that seclusion drives obesity, in both animal studies (single housing) [14] and in humans [45–48]. Our monkeys had access to social contact with at least one conspecific throughout the study, suggesting that the observed weight gain was largely due to WSD unlike earlier conclusions; but why did ImE increase “time alone?” Although somewhat counter-intuitive, spending time in close contact with conspecifics correlated with signs of depression in cynomolgus macaques [49]. Therefore, ImE could be antidepressive. Alternatively, it is possible that the observed increase in “time alone” has to do with their engagement in other activities, such as locomotion or vigilance (not to be confused with anxiety). Being in close social contact with another individual is incompatible with jumping about the pen (i.e., animals cannot engage in both behaviors simultaneously). Another potential reason for this finding is our method of observations (live for focal versus videotape for provoked tests). Had the observer, who was familiar to the monkeys, not been present, the monkeys may have behaved somewhat differently [50].

“Self-groom” was lower in the ImE group and decreased upon administration of DE to animals treated with placebo for 2 years. “Self-groom” is often considered a displacement behavior, indicative of anxiety similar to scratch and body shake [51] further suggesting that ImE decreased anxiety. Because “self-groom” was measured as a “duration,” but other anxiety behaviors (e.g., scratch) were measured as “frequency,” we were not able to combine these two behaviors into one larger anxiety category. Nonetheless, ImE-treated animals exhibited increased locomotion. One author postulated that pacing and other forms of stereotypy may be a coping mechanism for captive animals [52]. In the current study, there was no effect of time or treatment on stereotypic behavior. Taken together, these results suggest that ImE and DE helped the monkeys cope better with their environment by increasing activity.

Elevated anxiety in the placebo group was also indicated by behavior in the Human Intruder Test. That is, there was higher “locomotion” in the placebo-treated group during the period of acclimation to the test cage. This finding corroborates those of the focal observations and suggests that ImE might mitigate against anxiety. Excessive “freezing” while a potential threat (human intruder) is present, but has not yet noticed the subject, is also considered to be a sign of a fearful or anxious temperament [53]. However, there was no difference between treatment groups in “freezing” when exposed to the profile of a human intruder.

Therefore, both focal observations and HIT indicate that the placebo animals may have been more anxious. The lack of vigilance exhibited by the placebo group in the home pen could coexist with anxiety that manifests as self-grooming. Also of interest was the decrease in propensity to touch novel objects (e.g., decreased “Bold Score” on the novel object tests) after only one year, which was not different between treatment groups. Hence, it appears that WSD decreases boldness or exploratory behavior. Unfortunately, there was a lack of food motivation in attempts to train the monkeys in a Delayed Recall paradigm and in a Spatial Maze paradigm. Lack of food motivation may have also impacted their interest in the novel fruit.

Another aspect of boldness in humans is novelty seeking, which is inversely related to dopamine (DA) D2 receptors in the striatum. This inverse relationship is lost in addicts and obese subjects [54]. Drugs of abuse and palatable food with high-fat and high-sugar content activate DA reward circuitry [55]. However, in food addiction, we do not know the mechanism by which food activates the DA reward pathway [56]. We speculate that the decrease in boldness in our WSD-fed macaques may reflect decreased DA D2 receptors in the ventral tegmental area (VTA) in a manner similar to the action of drugs of abuse, although the mechanism remains a mystery.

Rhesus macaques are a relatively aggressive species, and aggression toward conspecifics in the pens was highest as social ranks were resolved to the satisfaction of the group. Changes in aggression have been reported in cynomolgus macaques with diet and destabilized groupings [57]. Elevated dyad reciprocal aggression was also observed in unstable social groups [58], and food size can increase aggression [59]. However, after social groups had formed in this study (each pen contained animals with different treatments), there was no statistical difference in the frequency of initiated aggression between ImE-, DE-, or placebo-treated monkeys. Overall aggression decreased by 50% from B0 to the 2.5-year time point, but this difference was not significant between treatments. The largest delta of decrease in aggression initiations occurred in the placebo animals, which equaled 0.086 initiations/min. Although attrition by year 2.5 precluded statistical significance, it appeared that aggression was the inverse of close social contact. This relationship has been observed in subordinate cynomolgus macaques as well [60].

Altogether, WSD had serious effects on important behaviors in rhesus macaques such as socialization, consumption, exercise, grooming, and boldness that may parallel behaviors observed in humans eating a typical American diet. “Close social,” “alone,” “consume,” and “locomote” were modified by ImE therapy, whereas boldness was not. ImE-treated WSD-fed animals exhibited an increase in the amount of “alone time” and in “locomote,” as well as a decrease in “close social” and “self-groom.” We attribute this constellation of behaviors to decreased anxiety relative to placebo-treated animals. In the provocation tests, the placebo group showed higher locomotion during the HIT acclimation period, a behavior that may further indicate anxiety in a different context. Clearly, much needed changes are needed in the American diet. In addition, the central neural and peripheral cellular and molecular mechanisms that lead to the results from eating a high fat-high sugar diet need resolution.

## Figures and Tables

**Table 1:** The group, treatment, and ranks of the animals at the start and end of the study.

Animal	Pen	Treatment	Rank	Rank
			Start	End
A1	1	Placebo	D	D
A2	1	ImE	S	∼
A3	1	Placebo	M	S
A4	1	Placebo	M	M
B1	2	ImE	D	D
B2	2	DE	M	M
B3	2	DE	S	S
B4	2	Placebo	M	∼
C1	3	DE	D	D
C2	3	Placebo	M	M
C3	3	ImE	S	S
C4	3	ImE	M	∼
D1	4	DE	M	S
D2	4	DE	S	D
D3	4	Placebo	D	∼
D4	4	ImE	M	∼
E1	5	ImE	M	M
E2	5	DE	D	D
E3	5	Placebo	S	S
F1	6	DE	M	∼
F2	6	ImE	S	D
F3	6	DE	D	S
F4	6	Placebo	M	∼
G1	7	Placebo	D	∼
G2	7	Placebo	S	S
G3	7	ImE	M	D
H1	8	ImE	S	S
H2	8	ImE	D	D

∼ denotes missing data, which by 30 months was largely due to attrition. D = dominant (highest ranking) monkey. M = midranking monkey(s). S = subordinate (lowest ranking) monkey. Please note that the last 4 animals (G 2-3, H1-2) were initially extras, but they were added after other animals were removed from the study.

**Table 2:** Time points for behavioral testing (numbers in parentheses are mean ± SD).

Time point	Description	Anxiety test performed
Baseline 0 (B0)	Approximately 2 (1.9 ± 0.7) months after introduction to the group; prior to introduction of WSD and ovariectomy	HIT NOT Focal observation
Baseline 1 (B1)	Approximately 3 (2.8 ± 0.6) months after B0; animals on high-fat diet; prior to ovohysterectomy	Focal observations
Year 1	Approximately 7.5 (7.5 ± 0.8) months after B1	HIT NOT Focal observations
Year 2	12 months after year 1	HIT NOT Focal observations
Year 2.5	Prior to end of study	HIT NOT Focal observations

**Table 3:** Ethogram of behaviors coded in the home cage assessments.

Behavioral Class	Behavior	Definition
Social behaviors (measured in percent of time)	Groom	Focal individual is picking at hair and/or skin of another individual (focal can initiate or receive behavior)
	Proximity	Focal individual is within arms length of another individual without touching
	Touch	Focal is in physical contact with another individual
	Ventral contact	Special case of touch, in which ventral surface of both animals are in contact
	Positive social behavior	Combined behavior which includes groom, touch, or ventral contact
	Alone	Focal individual is not in any social contact with other monkeys
Nonsocial behaviors (measured in percent of time)	Stereotypical behavior	Repetitive behavior with no apparent purpose, such as pacing, circling, or poking eye
	Consume	Handling and ingesting food and/or water
	Locomote	Movement (e.g., walk, run)
	Object play	Focal individual manipulates object (e.g., toys or structures in the corral) other than food
	Self-groom	Focal individual grooms self
	Sleep	Focal individual is sitting with eyes closed, usually huddled with other individuals
	Stationary	Focal individual sitting quietly, not engaged in other behavior
Events (measured as frequency)	Aggression	Bite, hit, slap
	Fear grimace	Focal individual bars teeth
	Lipsmack	Rapid movement of lips
	Scratch	Common usage
	Threat	Open mouth threat gesture (focal can initiate or receive behavior)
	Yawn	Common usage
	Dominance-related behavior	Combined behavior which includes aggression, chase, displace, and threat

**Table 4:** Ethogram of behaviors coded during the Human Intruder Test.

Behavior	Operational definition
Vigilant	Subject's gaze is not directed toward human intruder
Freeze	Tense body posture with no movement and no vocalization
Locomotion	Active behavior resulting in movement from original location (e.g., moving across cage)
Stationary	Focal individual sitting quietly, not engaged in other behavior
Stereotypical behavior	Repetitive behavior with no apparent purpose
Vocalizations	Includes coo and shriek
Yawn	Subject opens mouth very wide, baring upper teeth
Self-directed anxiety behavior	Subject scratches or shakes (quick action of rotating head and top of shoulders back and forth)

## Data Availability

The data used to support the findings of this study are available from the corresponding author upon request.

## Additional Points

(1) Old rhesus macaques were studied as a model of menopausal women who eat an obesogenic or “western-style diet” (WSD) and receive hormone therapy (HT) in the perimenopause or significantly later. (2) Focal and provoked behaviors were observed in a longitudinal manner over 2.5 years. (3) Animals received an empty Silastic capsule (placebo), an estradiol-filled capsule immediately upon ovohysterectomy (ImE), or an empty capsule for 2 years followed by an estradiol-filled capsule for 6 months (DE). (4) WSD increased consumption and decreased boldness, regardless of treatment. (5) Placebo-treated animals exhibited more sedentary and anxiety-type behaviors than ImE-treated animals. (6) DE-treated animals were similar to ImE animals in locomote and self-groom behaviors after 6 months, but not with respect to time alone or in close social.

## References

[1] Harman S. M., Brinton E. A., Clarkson T. (2004). Is the WHI relevant to HRT started in the perimenopause?. *Endocrine*.

[2] Harman S. M., Naftolin F., Brinton E. A., Judelson D. R. (2005). Is the estrogen controversy over? Deconstructing the Women’s Health Initiative study: a critical evaluation of the evidence. *Annals of the New York Academy of Sciences*.

[3] Schmidt P. J., Rubinow D. R. (2009). Sex hormones and mood in the perimenopause. *Annals of the New York Academy of Sciences*.

[4] Shanmugan S., Epperson C. N. (2012). Estrogen and the prefrontal cortex: towards a new understanding of estrogen’s effects on executive functions in the menopause transition. *Human Brain Mapping*.

[5] Epperson C. N., Amin Z., Ruparel K., Gur R., Loughead J. (2012). Interactive effects of estrogen and serotonin on brain activation during working memory and affective processing in menopausal women. *Psychoneuroendocrinology*.

[6] Hodis H. N., Mack W. J., Henderson V. W. (2016). Vascular effects of early versus late postmenopausal treatment with estradiol. *New England Journal of Medicine*.

[7] Foster R. H., Balfour J. A. (1997). Estradiol and dydrogesterone. A review of their combined use as hormone replacement therapy in postmenopausal women. *Drugs & Aging*.

[8] Stipic I., Polasek O., Vulic M., Punda H., Grandic L., Strinic T. (2012). Estrogen replacement therapy improves pulmonary function in postmenopausal women with genital prolapse. *Rejuvenation Research*.

[9] Engelmann F., Rivera A., Park B., Messerle-Forbes M., Jensen J. T., Messaoudi I. (2016). Impact of estrogen therapy on lymphocyte homeostasis and the response to seasonal influenza vaccine in post-menopausal women. *PLoS One*.

[10] Christianson M. S., Mensah V. A., Shen W. (2015). Multiple sclerosis at menopause: potential neuroprotective effects of estrogen. *Maturitas*.

[11] Smyth S., Heron A. (2006). Diabetes and obesity: the twin epidemics. *Nature Medicine*.

[12] Toth M. J., Tchernof A., Sites C. K., Poehlman E. T. (2006). Menopause-related changes in body fat distribution. *Annals of the New York Academy of Sciences*.

[13] Jull J., Stacey D., Beach S. (2014). Lifestyle interventions targeting body weight changes during the menopause transition: a systematic review. *Journal of Obesity*.

[14] Yue F., Zhang G., Quintero J. E., Gash D. M., Zhang Z. (2017). Role of social interaction, exercise, diet, and age on developing and untreated diabetes in cynomolgus monkeys. *Experimental Gerontology*.

[15] Kaplan J. R., Adams M. R., Clarkson T. B., Manuck S. B., Shively C. A. (1991). Social behavior and gender in biomedical investigations using monkeys: studies in atherogenesis. *Laboratory Animal Science*.

[16] Bethea C. L., Mueller K., Reddy A. P., Kohama S. G., Urbanski H. F. (2017). Effects of obesogenic diet and estradiol on dorsal raphe gene expression in old female macaques. *PLoS One*.

[17] Urbanski H. F., Mueller K., Bethea C. L. (2017). Effect of an obesogenic diet on circadian activity and serum hormones in old monkeys. *Endocrine Connections*.

[18] Purnell J. Q., Bethea C. L. Long-term western style diet (WSD) blocks the beneficial metabolic effects of immediate estradiol (E) replacement in older surgically menopausal macaques.

[19] Purnell J. Q., Bethea C. L. Benefits of immediate versus delayed estradiol replacement on body composition and glucose metabolism in older surgically menopausal macaques on a long-term western style diet.

[20] Purnell J. Q., Urbanski H. F., Bethea C. L. Effect of estrogen on body composition, activity, and hormone levels in non-human primates on a western-style diet.

[21] Yamada T., Press M., Vesselinovitch D., Wissler R. W. (1988). Quantitative ultrastructural analysis of coronary atherosclerotic involvement in two macaque species. *Experimental and Molecular Pathology*.

[22] Uno H., Poff B. (1983). Coronary arterial ectasia, a predominant type of coronary sclerosis in aged captive rhesus monkeys (*Macaca mulatta*). *American Journal of Pathology*.

[23] Bond M. G., Bullock B. C., Bellinger D. A., Hamm T. E. (1980). Myocardial infarction in a large colony of nonhuman primates with coronary artery atherosclerosis. *American Journal of Pathology*.

[24] Coleman K., Pierre P. J. (2014). Assessing anxiety in nonhuman primates. *ILAR Journal*.

[25] Altmann J. (1974). Observational study of behavior: sampling methods. *Behaviour*.

[26] Kalin N. H., Shelton S. E. (1989). Defensive behaviors in infant rhesus monkeys: environmental cues and neurochemical regulation. *Science*.

[27] Coleman K., Schapiro S. J. (2017). Individual differences in temperament and behavioral management. *The Handbook of Primate Behavioral Management*.

[28] Hannibal D. L., Bliss-Moreau E., Vandeleest J., McCowan B., Capitanio J. (2017). Laboratory rhesus macaque social housing and social changes: Implications for research. *American Journal of Primatology*.

[29] Reinhardt V. (1991). Group formation of previously single-caged adult rhesus macaques for the purpose of environmental enrichment. *Journal of Experimental Animal Science*.

[30] Truelove M. A., Martin A. L., Perlman J. E., Wood J. S., Bloomsmith M. A. (2017). Pair housing of macaques: a review of partner selection, introduction techniques, monitoring for compatibility, and methods for long-term maintenance of pairs. *American Journal of Primatology*.

[31] Meishvilli N. V., Chalyan V. G., Rozhkova Y. Y. (2009). The causes of intragroup aggression in rhesus macaques. *Neuroscience and Behavioral Physiology*.

[32] Choi Y., Ahn K.-H., Lee J.-I. (2014). Suppurative bite wound by repetitive aggression of dominance hierarchy during group housing in rhesus monkeys. *Laboratory Animal Research*.

[33] Fairbanks L. A., McGuire M., Kerber W. (1978). Effects of group size, composition, introduction technique and cage apparatus on aggression during group formations in rhesus monkeys. *Psychological Reports*.

[34] Crockett C. M., Bowers C. L., Shimoji M. (1995). Behavioral responses of longtailed macaques to different cage sizes and common laboratory experiences. *Journal of Comparative Psychology*.

[35] Kaplan J. R., Manuck S. B. (1999). Status, stress, and atherosclerosis: the role of environment and individual behavior. *Annals of the New York Academy of Sciences*.

[36] Shufelt C. L., Johnson B. D., Berga S. L. (2011). Timing of hormone therapy, type of menopause, and coronary disease in women: data from the national heart, lung, and blood institute-sponsored women’s ischemia syndrome Evaluation. *Menopause*.

[37] Rocca W. A., Grossardt B. R., Shuster L. T. (2010). Oophorectomy, menopause, estrogen, and cognitive aging: the timing hypothesis. *Neurodegenerative Diseases*.

[38] Johnson Z. P., Lowe J., Michopoulos V., Moore C. J., Wilson M. E., Toufexis D. (2013). Oestradiol differentially influences feeding behaviour depending on diet composition in female rhesus monkeys. *Journal of Neuroendocrinology*.

[39] Sullivan E. L., Shearin J., Koegler F. H., Cameron J. L. (2012). Selective estrogen receptor modulator promotes weight loss in ovariectomized female rhesus monkeys (*Macaca mulatta*) by decreasing food intake and increasing activity. *American Journal of Physiology-Endocrinology and Metabolism*.

[40] Debnath M., Agrawal S., Agrawal A., Dubey G. P. (2016). Metaflammatory responses during obesity: pathomechanism and treatment. *Obesity Research and Clinical Practice*.

[41] Divella R., De Luca R., Abbate I., Naglieri E., Daniele A. (2016). Obesity and cancer: the role of adipose tissue and adipo-cytokines-induced chronic inflammation. *Journal of Cancer*.

[42] Miller A. P., Chen Y. F., Xing D., Feng W., Oparil S. (2003). Hormone replacement therapy and inflammation: interactions in cardiovascular disease. *Hypertension*.

[43] Nosalski R., Guzik T. J. (2017). Perivascular adipose tissue inflammation in vascular disease. *British Journal of Pharmacology*.

[44] Vural P., Akgul C., Canbaz M. (2006). Effects of hormone replacement therapy on plasma pro-inflammatory and anti-inflammatory cytokines and some bone turnover markers in postmenopausal women. *Pharmacological Research*.

[45] Horsten M., Mittleman M. A., Wamala S. P., Schenck-Gustafsson K., Orth-Gomer K. (1999). Social relations and the metabolic syndrome in middle-aged Swedish women. *European Journal of Cardiovascular Risk*.

[46] Pantell M., Rehkopf D., Jutte D., Syme S. L., Balmes J., Adler N. (2013). Social isolation: a predictor of mortality comparable to traditional clinical risk factors. *American Journal of Public Health*.

[47] Sun M., Choi E. Y., Magee D. J., Stets C. W., During M. J., Lin E. J. (2014). Metabolic effects of social isolation in adult C57BL/6 mice. *International Scholarly Research Notices*.

[48] Papadopoulos S., Brennan L. (2015). Correlates of weight stigma in adults with overweight and obesity: a systematic literature review. *Obesity*.

[49] Shively C. A., Register T. C., Friedman D. P., Morgan T. M., Thompson J., Lanier T. (2005). Social stress-associated depression in adult female cynomolgus monkeys (*Macaca fascicularis*). *Biological Psychology*.

[50] Iredale S. K., Nevill C. H., Lutz C. K. (2010). The influence of observer presence on baboon (*Papio* spp.) and rhesus macaque (*Macaca mulatta*) behavior. *Applied Animal Behaviour Science*.

[51] Kutsukake N., Castles D. L. (2001). Reconciliation and variation in post-conflict stress in Japanese macaques (*Macaca fuscata fuscata*): testing the integrated hypothesis. *Animal Cognition*.

[52] Poirier C., Bateson M. (2017). Pacing stereotypies in laboratory rhesus macaques: implications for animal welfare and the validity of neuroscientific findings. *Neuroscience and Biobehavioral Reviews*.

[53] Kalin N. H., Shelton S. E., Rickman M., Davidson R. J. (1998). Individual differences in freezing and cortisol in infant and mother rhesus monkeys. *Behavioral Neuroscience*.

[54] Savage S. W., Zald D. H., Cowan R. L. (2014). Regulation of novelty seeking by midbrain dopamine D2/D3 signaling and ghrelin is altered in obesity. *Obesity*.

[55] Michaelides M., Thanos P. K., Volkow N. D., Wang G. J. (2012). Translational neuroimaging in drug addiction and obesity. *ILAR Journal*.

[56] Baik J. H. (2013). Dopamine signaling in food addiction: role of dopamine D2 receptors. *BMB Reports*.

[57] Kaplan J. R., Manuck S. B., Shively C. (1991). The effects of fat and cholesterol on social behavior in monkeys. *Psychosomatic Medicine*.

[58] Beisner B. A., Jin J., Fushing H., McCowan B. (2015). Detection of social group instability among captive rhesus macaques using joint network modeling. *Current Zoology*.

[59] Mathy J. W., Isbell L. A. (2001). The relative importance of size of food and interfood distance in eliciting aggression in captive rhesus macaques (*Macaca mulatta*). *Folia Primatologica*.

[60] Shively C. A. (1998). Social subordination stress, behavior and central monoaminergic function in female cynomolgus monkeys. *Biological Psychiatry*.

